# Supplementary material for: The Systems Biology Research Tool: evolvable open-source software
Source: BMC Syst Biol. 2008 Jun 29;2:55. doi: 10.1186/1752-0509-2-55 (PMC2446383; doi:10.1186/1752-0509-2-55)
Supplement: Additional file 1 — SBRT Archive. An archive of the current version of the Systems Biology Research Tool. [file 1752-0509-2-55-S1.zip › sbrt-1.4.0/doc/developers_guide/api/index-files/index-8.html]

H-Index


|  |  |  |
| --- | --- | --- |
| **Overview** \| Package \| Class \| Use \| **Tree** \| **Deprecated** \| **Index** \| **Help** | |  |
| **PREV LETTER**   **NEXT LETTER** | **FRAMES**    **NO FRAMES**     **All Classes** |  |


A B C D E F G H I J K L M N O P Q R S T U V W X Y Z 

---


## **H**

**Hamming** - Class in sbrt.kernel.math.geom.comp: This class is used to compute Hamming distances - the number of differences between two "strings" of equal length. **Hamming()** - Constructor for class sbrt.kernel.math.geom.comp.Hamming: **hasAllVariables(Collection<?>)** - Method in class sbrt.kernel.math.AbstractLinearComb.Term: Indicates if this term contains all of the variables in the provided collection. **hasAllVariables(Collection<?>)** - Method in class sbrt.kernel.math.HashLinearComb: Indicates if this mathematical expression contains all of the variables in the provided collection. **hasAllVariables(Collection<?>)** - Method in interface sbrt.kernel.math.MathExpr: Indicates if this mathematical expression contains all of the variables in the provided collection. **hasAllVariables(Collection<?>)** - Method in interface sbrt.kernel.math.Polynomial.Term: Indicates if this term contains all of the variables in the provided collection. **HASH** - Static variable in class sbrt.shell.text.SpecialChars: # **hashCode()** - Method in class sbrt.kernel.fba.AbstractRxn: Returns a content-based hash code for this reaction. **hashCode()** - Method in interface sbrt.kernel.fba.ChemRxn: Returns a content-based hash code for this chemical reaction. **hashCode()** - Method in class sbrt.kernel.fba.ExchangeRxn: Returns a content-based hash code for this exchange reaction. **hashCode()** - Method in class sbrt.kernel.math.AbstractLinearComb.Term: Returns a content-based hash code for this term. **hashCode()** - Method in class sbrt.kernel.math.AbstractPolynomial: Returns a content-based hash code of this polynomial. **hashCode()** - Method in class sbrt.kernel.math.algebra.HashMatrix: Returns a content-based hash code for this matrix. **hashCode()** - Method in class sbrt.kernel.math.algebra.IndexPair: Returns a content-based hash code. **hashCode()** - Method in interface sbrt.kernel.math.algebra.Matrix: Returns a content-based hash code for this matrix. **hashCode()** - Method in class sbrt.kernel.math.algebra.MatrixWrap: Returns a content-based hash code for this matrix. **hashCode()** - Method in class sbrt.kernel.math.algebra.RowOrTreeMatrix: Returns a content-based hash code for this matrix. **hashCode()** - Method in class sbrt.kernel.math.algebra.TreeMatrix: Returns a content-based hash code for this matrix. **hashCode()** - Method in class sbrt.kernel.math.geom.ParallelHyperplanes: Returns a content-based hash code. **hashCode()** - Method in class sbrt.kernel.math.graph\_theory.DirectedGraph.Edge: Returns a content-based hash code for this directed edge. **hashCode()** - Method in class sbrt.kernel.math.graph\_theory.Path: Returns a hash code based on the nodes contained in this path. **hashCode()** - Method in interface sbrt.kernel.math.LinearComb: Returns a content-based hash code for this linear combination. **hashCode()** - Method in interface sbrt.kernel.math.MathExpr: Returns a content-based hash code for this mathematical expression. **hashCode()** - Method in interface sbrt.kernel.math.Polynomial: Returns a content-based hash code for this polynomial. **hashCode()** - Method in interface sbrt.kernel.math.Polynomial.Term: Returns a content-based hash code for this term. **hashCode()** - Method in class sbrt.kernel.util.BooleanRelation: **hashCode()** - Method in class sbrt.kernel.util.UnmodifiableCollection: See Collection.hashCode(). **hashCode()** - Method in class sbrt.kernel.util.UnmodifiableList: See List.hashCode(). **hashCode()** - Method in class sbrt.kernel.util.UnmodifiableMap: See Map.hashCode(). **hashCode()** - Method in class sbrt.kernel.util.UnmodifiableSet: See Set.hashCode(). **HashConvexPolytope**<V> - Class in sbrt.kernel.math.geom: This class is a concrete implemenation of `ConvexPolytope`. **HashConvexPolytope(Set<ParallelHyperplanes<V>>)** - Constructor for class sbrt.kernel.math.geom.HashConvexPolytope: Constructs a new convex poltyope from the provided linear system solution and variable bounds. **HashConvexPolytopeOld**<V> - Class in sbrt.kernel.math.geom: This class is a concrete implemenation of `ConvexPolytope`. **HashConvexPolytopeOld(LinearSystemSolution<V>, Map<? extends V, Interval>, double)** - Constructor for class sbrt.kernel.math.geom.HashConvexPolytopeOld: Constructs a new convex poltyope from the provided linear system solution and variable bounds. **HashLinearComb**<V> - Class in sbrt.kernel.math: This class is a concrete implemenation of `LinearComb`. **HashLinearComb(LinearComb.Term<? extends V>[], double, double)** - Constructor for class sbrt.kernel.math.HashLinearComb: Constructs a new linear combination from the provided terms and constant. **HashLinearComb(Collection<? extends LinearComb.Term<? extends V>>, double, double)** - Constructor for class sbrt.kernel.math.HashLinearComb: Constructs a new linear combination from the provided terms and constant. **HashLinearComb.Term**<V> - Class in sbrt.kernel.math: This class is a concrete implementation of `LinearComb.Term`. **HashLinearComb.Term(double, V)** - Constructor for class sbrt.kernel.math.HashLinearComb.Term: Constructs a new linear combination term with the provided coefficient and variable. **HashLinearComb.Term(V, double)** - Constructor for class sbrt.kernel.math.HashLinearComb.Term: Constructs a new linear combination term with the provided coefficient and variable. **HashLinearComb.Term(V)** - Constructor for class sbrt.kernel.math.HashLinearComb.Term: Constructs a new linear combination term with the provided variable. **HashLinearSystemSolution**<V> - Class in sbrt.kernel.math.algebra: This class is a concrete implemenation of `LinearSystemSolution`. **HashLinearSystemSolution(Map<? extends V, ? extends LinearComb<? extends V>>)** - Constructor for class sbrt.kernel.math.algebra.HashLinearSystemSolution: Constructs a new hash code-based linear system solution. **HashMatrix**<R,C> - Class in sbrt.kernel.math.algebra: This class is a `java.util.LinkedHashMap` based implementation of `SmdMatrix`. **HashMatrix()** - Constructor for class sbrt.kernel.math.algebra.HashMatrix: Constructs an emtpy `HashMatrix` with the default initial capacity (16) and the default load factor (0.75). **HashMatrix(int, int, float)** - Constructor for class sbrt.kernel.math.algebra.HashMatrix: Constructs an empty `HashMatrix` with the provided initial capacities and load factor. **hasNext()** - Method in class sbrt.kernel.util.UnmodifiableIterator: See Iterator.hasNext(). **hasNext()** - Method in class sbrt.shell.io.ComiFileSource: Indicates if this iterator can return another file using the `next()` method . **hasNext()** - Method in class sbrt.shell.io.ComoFileSource: Indicates if this iterator can return another file using the `next()` method . **hasNext()** - Method in class sbrt.shell.io.fba.CofoofSource: Indicates if this iterator can return another file using the `next()` method . **hasNext()** - Method in class sbrt.shell.io.fba.RofoofSource: Indicates if this iterator can return another file using the `next()` method . **hasNext()** - Method in class sbrt.shell.io.fba.SfoofSource: Indicates if this iterator can return another file using the `next()` method . **hasNext()** - Method in class sbrt.shell.io.RomiFileSource: Indicates if this iterator can return another file using the `next()` method . **hasNext()** - Method in class sbrt.shell.io.RomoFileSource: Indicates if this iterator can return another file using the `next()` method . **hasNext()** - Method in class sbrt.shell.io.SerializedFileIterator: Indicates if the file has more serialized objects. **hasNext()** - Method in class sbrt.shell.io.SmoFileSource: Indicates if this iterator can return another file using the `next()` method . **hasNext()** - Method in class sbrt.shell.io.TextFileLineIterator: Indicates if the file has more lines. **hasValueFor(Object)** - Method in class sbrt.shell.io.ProcessInputFile: Indicates if a value exists for the provided key in this manager input. **hasValueFor(Object)** - Method in interface sbrt.shell.mng.ManagerInput: Indicates if a value exists for the provided key in this manager input. **hasValueFor(Object)** - Method in class sbrt.shell.mng.MapManagerInput: Indicates if a value exists for the provided key in this manager input. **hasVariable(Object)** - Method in class sbrt.kernel.math.AbstractLinearComb.Term: Indicates if the provided object is a variable contained in this term. **hasVariable(Object)** - Method in class sbrt.kernel.math.HashLinearComb: Indicates if the provided object is a variable contained in this mathematical expression. **hasVariable(Object)** - Method in interface sbrt.kernel.math.MathExpr: Indicates if the provided object is a variable contained in this mathematical expression. **hasVariable(Object)** - Method in interface sbrt.kernel.math.Polynomial.Term: Indicates if the provided object is a variable contained in this term. **hasVariables()** - Method in class sbrt.kernel.math.AbstractLinearComb.Term: Returns `true`. **hasVariables()** - Method in class sbrt.kernel.math.HashLinearComb: Indicates if this mathematical expression contains any variables. **hasVariables()** - Method in interface sbrt.kernel.math.MathExpr: Indicates if this mathematical expression contains any variables.

---


|  |  |  |
| --- | --- | --- |
| **Overview** \| Package \| Class \| Use \| **Tree** \| **Deprecated** \| **Index** \| **Help** | |  |
| **PREV LETTER**   **NEXT LETTER** | **FRAMES**    **NO FRAMES**     **All Classes** |  |


A B C D E F G H I J K L M N O P Q R S T U V W X Y Z 

---
